# Accounting for the genetic load in assisted reproductive technology

**DOI:** 10.1002/ctm2.864

**Published:** 2022-05-25

**Authors:** Cock van Oosterhout, Daniel Marcu, Simone Immler

**Affiliations:** ^1^ School of Environmental Sciences University of East Anglia Norwich UK; ^2^ School of Biological Sciences University of East Anglia Norwich UK

**Keywords:** assisted reproductive technology, deleterious mutations, genetic load, human fertility

## Abstract

The genetic load in the human genome has important ramifications for assisted reproductive technology (ART), human reproduction and fertility more generally. Here, we discuss these topics in the light of evolutionary genetic theory, the technological revolution in ART and the advances in the fields of genomics and bioinformatics.

## 1 HISTORICAL BACKGROUND

Nearly 44 years after the birth of the world's first ‘test‐tube baby’, over eight million babies have been conceived globally as the result of assisted reproductive technology (ART).^1^ Despite many medical and technological advances, the life‐birth rate per embryo transfer for *in vitro* fertilisation (IVF) currently stands at 32% for couples of a young reproductive age. This low rate is partly due to the high genetic load in the human genome. Selection against the genetic load might contribute to up to nearly 90% mortality of zygotes on average.

Mutations form the substrate of genetic variation that enables species to continue to evolve. However, the majority of the mutations that affect fitness are deleterious rather than beneficial, and they reduce fitness.^2^ Recent advances in genetics of humans, model‐animals and comparative genomics enable us to study the genetic load at the molecular level.^3^

Whole genome sequencing studies show that on average, a human may carry over a thousand deleterious mutations, including ∼250–300 loss‐of‐function mutations (1000 Genomes Project Consortium 2010), as well as fitness‐reducing variants at both coding and non‐coding sites.^4^ Generally, this genetic load can be tolerated by individuals as these deleterious mutations tend to be rare in populations, which means they are rarely expressed in homozygote condition. These mutations form part of the so called ‘masked load’.^3^ In contrast to this masked load, the realised load of deleterious mutations does reduce the fitness of individuals.^3^

Haldane developed theory to calculate how much fitness is lost due to the constant input of deleterious de novo mutations.^5^ Briefly, the genome‐wide rate of deleterious mutation rate (*U*) is the product of the neutral mutation rate per generation (*μ*), the number of bases in the diploid genome and the fraction that is selectively constrained (*C*) (see^6^). Haldane argued that whilst selection is removing deleterious mutations, new mutations enter the populations every generation.^5^ This creates an equilibrium between mutation and selection, and the equilibrium fitness ($\hat{W}$) in a population with a genome‐wide rate of deleterious mutations (*U*) equals $\hat{W}=e^{-U}$.

## 2 GENETIC LOAD IN HUMANS

**FIGURE 1:**
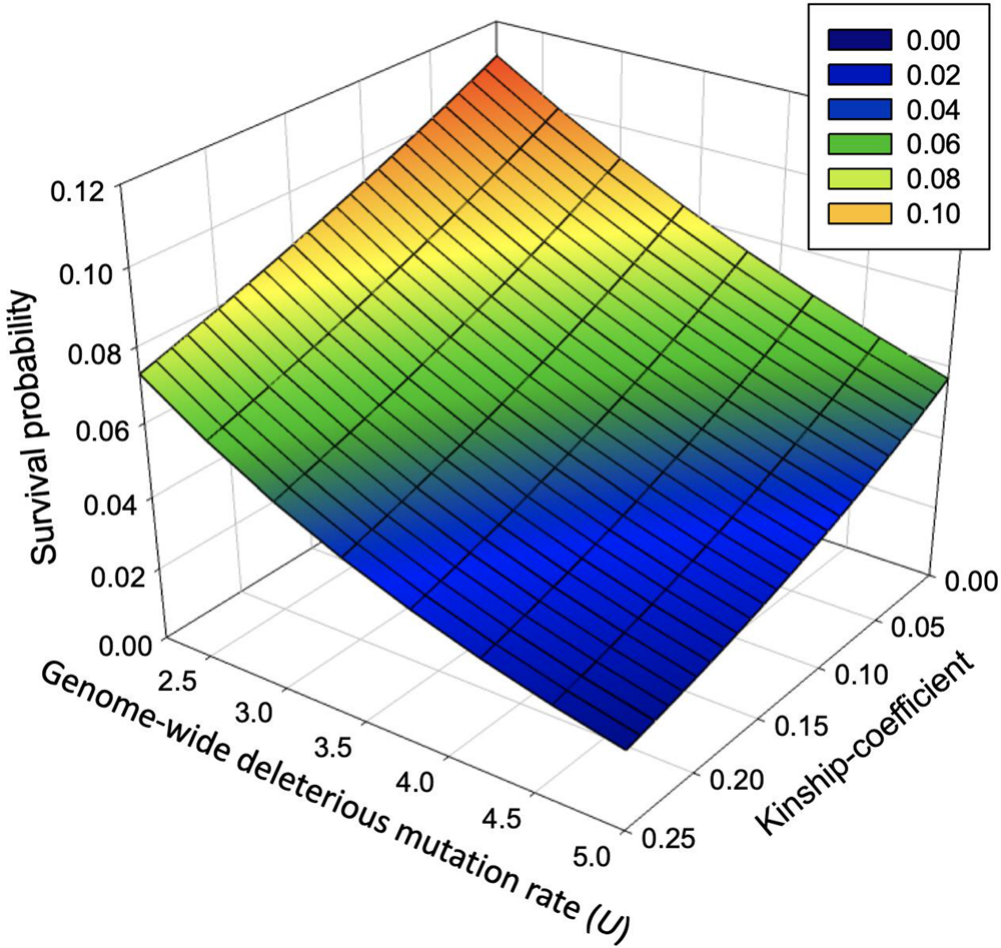
Survival probability per zygote (as a proxy for fertility) as a function of the genome‐wide deleterious mutation rate (*U*) and kinship coefficient of the parents in a population that is in a mutation‐selection equilibrium. Assuming *U *= 2.2, completely unrelated parents and hard selection, approximately 11% of zygotes are expected to make it to adulthood as the result of ‘selective deaths’ caused by the genetic load. This percentage decreases steeply with increased *U* and kinship coefficient (see ref. ^[^ ^3^ ^]^ for equations)

Estimates for *U* vary widely across species, and they seem to be particularly high for humans. Every generation, ∼70 new mutations arise in the human diploid genome.^7^ Assuming that at least 5% of the genome is under functional constraint, each genome accumulates several new deleterious mutations every generation. Keightley^7^ estimated that in humans *U* = 2.2, which suggests that the equilibrium fitness in humans equals $\hat{W}=e^{-2.2}\approx .11$. This means that an average human would have only 11% of the fitness of a ‘perfect’ individual without any deleterious mutations. In other words, from the moment of conception, an average human zygote would have around 11% probability to survive to adulthood, assuming hard selection (see below). With a higher kinship of the parents, or a higher genetic load, the realised load increases further, which leads to a steep drop in survival probability (Figure 1).

How do humans with relatively low reproductive capacity manage to persist with such a high genetic load? Some argued that the effects of mutations may exacerbate each other (i.e., synergistic epistasis), which increases the efficacy of purifying selection.^8^ It is also possible that by removing the genotypes with highest load, truncating selection can more efficiently eliminate mutations.^9^ Others argued that rather than purifying, selection may be stabilizing, which means that the effects of deleterious mutations can compensate each other. In that case, the mean trait value in the population is kept close to its optimal value resulting in a relatively low genetic load.^10^

Another plausible explanation is that soft selection could reduce the genetic load without impacting the population size.^11^ Soft selection is both density and frequency dependent. This simply means that it only removes the ‘excess’ of individuals that have a relatively low fitness. This excess consists of individuals who would have failed to survive or reproduce otherwise, for example because of limited resources. As such, soft selection does not determine how many individuals survive, but rather, it determines who survives.^11^ In contrast, hard selection is independent of the number of individuals in the population, or the frequency of other (superior or inferior) genotypes. With hard selection, there is no interaction between individuals, and survival is solely dependent on the absolute fitness of the individual (e.g., its realised load).^3^

## 3 SELECTION AGAINST THE GENETIC LOAD IN HUMANS

Both hard and soft selection operate against the realised load, and they may be particularly efficient during early development. Each female can produce many zygotes over her lifetime, and this offers many opportunities for hard selection. Although those selection‐events largely escape detection,^12^ the relatively high rate of spontaneous abortions of between 10% and 20% suggests that considerable selection operates in early development in humans. Such selection events could be instrumental for our species, enabling us to cope with a much higher genetic load than otherwise would be possible. Soft selection might also be able to differentiate among cells during the development of a multicellular individual, favouring mutations that are beneficial to the cells and preventing the spread of deleterious mutations.^13^

In addition, soft selection might operate at the gametic stage, particularly at sperm level.^14^ Emerging evidence suggests that gene products in mammalian sperm are transferred across spermatid cytoplasmic bridges, but the products of many genes are not completely shared.^15^ Such genes are known as ‘genoinformative markers’ (GIMs), and selection could operate very efficiently in sperm against recessive deleterious mutations at these GIMs. In fact, selection within an ejaculate has been shown to have major fitness consequences for the following generations.^16^ As such, selection at the haploid gametic stage would facilitate a considerable amount of soft selection.

## 4 RECENT CHANGES IN EVOLUTIONARY FORCES

Selection against our realised load has dramatically changed in recent times. Medical intervention has relaxed natural selection, allowing more mutations to accumulate. This is likely to result in a gradual increase in the genetic load, which reduces fertility (Figure 1). In addition, the total sperm count has plummeted by 59.3% between 1973 and 2011 with no evidence of improvements in recent years.^17^ This continuing decline in fertility, increased exposure to mutagens in our environment and the shift towards reproduction at later ages increase the mutation rate. Some scientists have warned that this poses a long‐term threat to the viability of humans.^18^, ^19^ The recent changes in mutation rate and selection in humans do not pose an immediate threat, as long as their effects on the genetic load can be countered by advances in medical technology, and in particular, advances in ART.

## 5 IMPACT OF ART ON GENETIC LOAD

Technologies such as IVF treatment and intracytoplasmic sperm injection (ICSI) largely bypass natural selection within the *in vivo* environment of the female reproductive tract.^20^ Cases where severe male infertility is treated with testicular sperm could bypass natural selection even further. In such cases, sperm cells that have not yet reached full maturity and acquired fertilisation capacity are used for reproduction. This eliminates many (if not all) selection steps that would naturally occur in the male reproductive tract prior to ejaculation and in the female reproductive tract following insemination. Potentially, such relaxed selection risks increasing the genetic load of offspring when compared to natural conception. In fact, studies suggest that children born to IVF are at potential risk of increased incidence of metabolic, cardiovascular and neurological disorders^21^, ^22^ and even early neonatal mortality.^23^ Therefore, future efforts should focus on improving and developing ART methods that mimic natural conception and its selection mechanisms.

## 6 FUTURE DEVELOPMENTS IN ART

We are approaching half a century since the first success story in ART, and yet we still know surprisingly little about the genomic processes involved in natural conception. This lack of knowledge is not least reflected in the persistently low success rates that are typical for most ARTs. The fact that most sperm cells never reach the site of fertilisation and that a large number of embryos never develop further than the first few cell divisions suggests that selection at many stages during natural conception is strong. All these selective stages are at least partly, if not completely, omitted during most ARTs, which may explain why the rate of live births is so low. Given the technological revolution and the advances in the field of genomics we have seen over the past 10 years or so, we are now in a position where we can address many unanswered questions around human reproduction and fertility. Harnessing these new technologies promises to improve ARTs and its success rates dramatically. Based on the purely theoretical evolutionary genetic arguments outlined above, continued ethical and scientific debate about research and development of ARTs is crucial. Such a conversation would not only help scientists to better gauge the possible consequences of ART and find solutions to address the current shortcomings of many of these technologies, but also benefit many future families relying on ART.

## CONFLICT OF INTEREST

The authors declare no competing interests.
